# Supplementary material for: Characterizing the nuclease accessibility of DNA in human cells to map higher order structures of chromatin
Source: Nucleic Acids Res. 2018 Nov 28;47(3):1239–54. doi: 10.1093/nar/gky1203 (PMC6379673; doi:10.1093/nar/gky1203)
Supplement: Supplementary Data [file gky1203_supplemental_files.pdf]

Supplementary Data

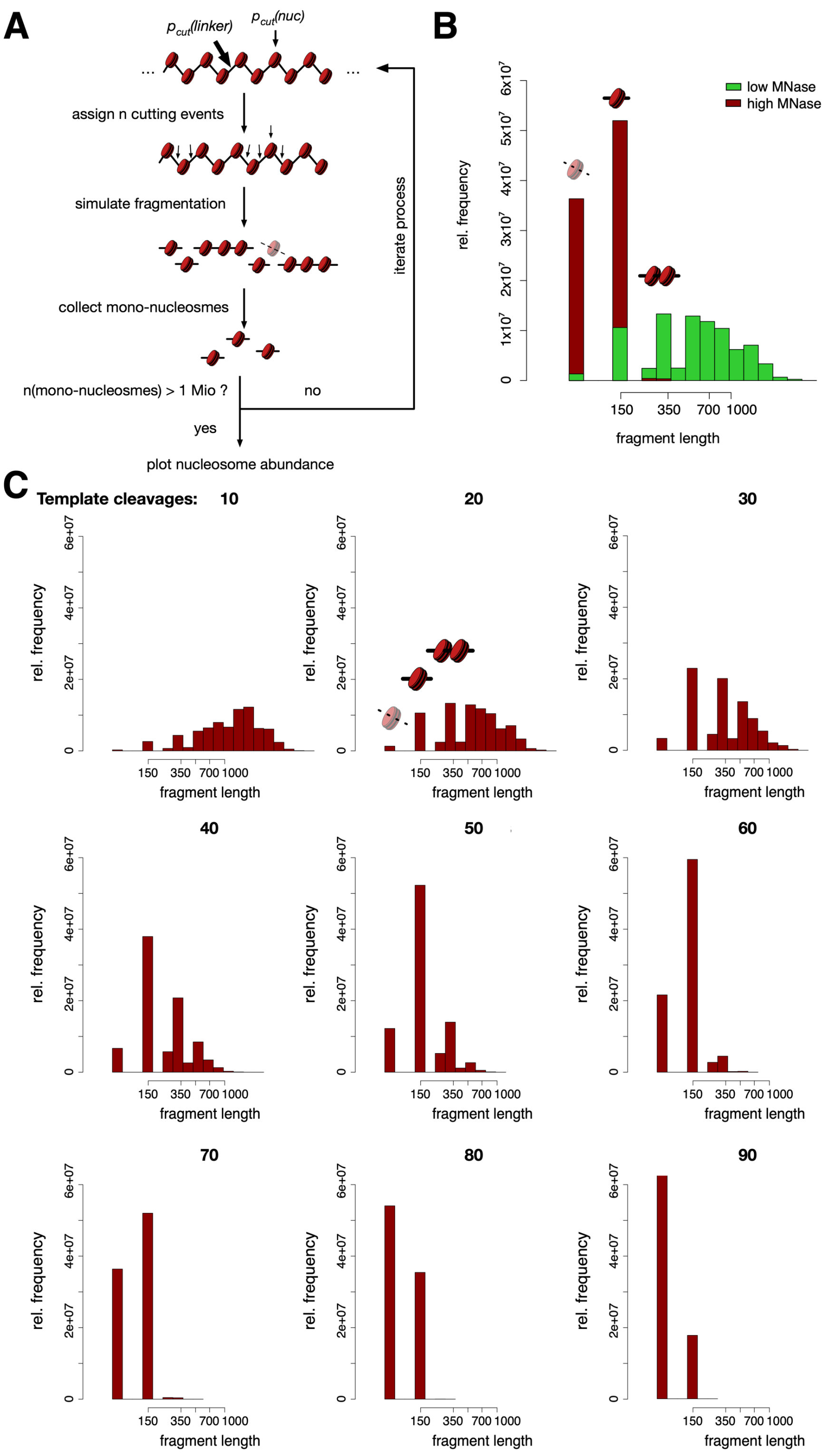

Supplementary Figure S1. Simulation of MNase digestions. (**A**) Schematic overview of the steps performed to simulate MNase digestions *in silico*. Different cleavage probabilities are assigned to inter- (p_{cut}(linker)) and intra-nucleosomal (p_{cut}(nuc)) sites. N sites were stochastically chosen and accordingly the nucleosomal array was fragmented. Mono-nucleosomes were collected, and the process was iterated until 1 Mio mono-nucleosomes have been collected. (**B-C**) Fragment length distribution of all fragments after the simulation. The frequency of the fragments was multiplied by the fragment length to simulate the intensities of DNA staining on agarose gels. (**B**) 70 template cleavages for high MNase and 20 cleavages for low MNase were used. (**C**) The number of template cleavages was simulated over a range of 10 to 90.

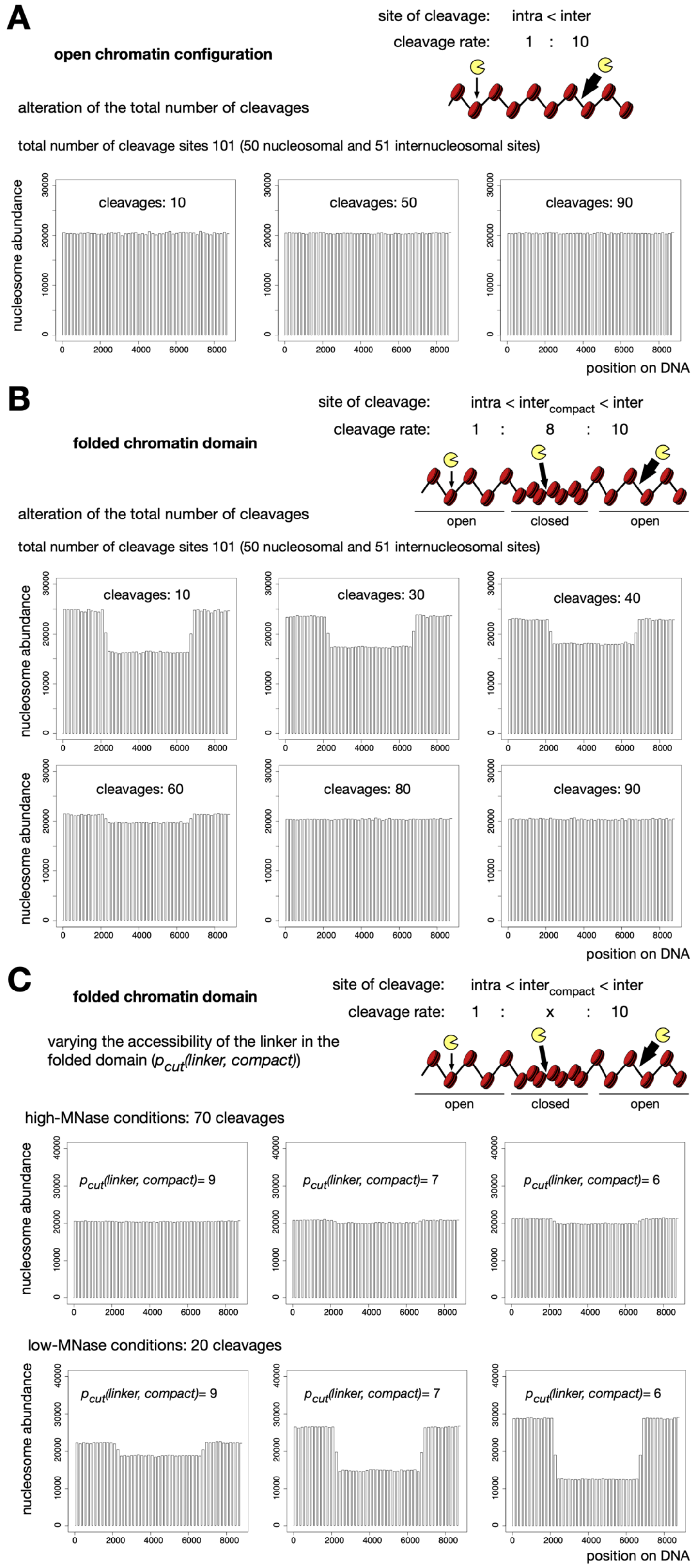

Supplementary Figure S2. Simulation of MNase digestions of nucleosomal arrays with varying parameters of domain accessibility. (**A**) A varying number of template cleavages was simulated on a nucleosomal array with equally accessible linker DNA (p_{cut}(linker)=constant). (**B**) Simulations, modelling nucleosomal arrays with a central compacted chromatin domain (p_{cut}(linker, compact) < p_{cut}(linker)). The number of cleavages was varied from 10 to 90. (**C**) A simulation, modelling the MNase hydrolysis of a nucleosomal array with a central compacted domain, as shown in (**B**), but the accessibility of the DNA linker in the compact domain (p_{cut}(linker, compact)) was varied as indicated.

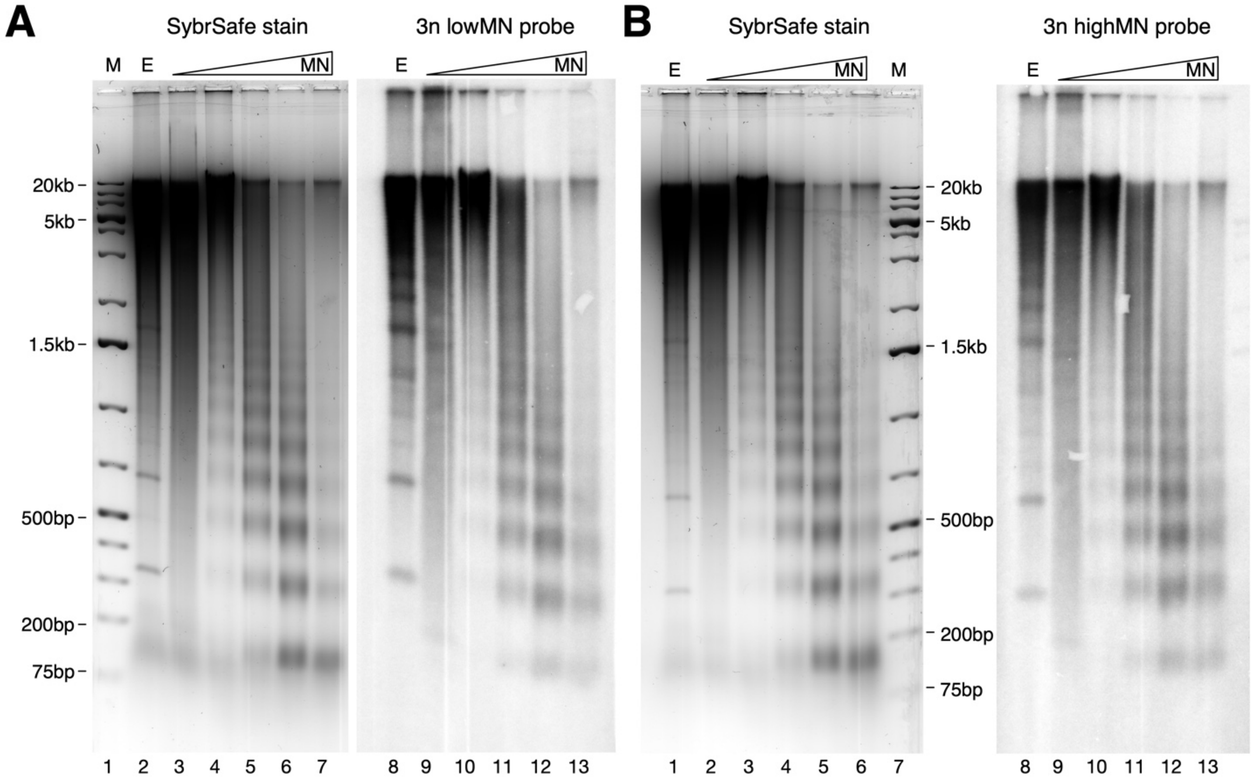

Supplementary Figure S3. Southern blots of MNase treated chromatin hybridized with isolated tri-nucleosomal DNA. (**A**) Purified DNA of HeLa chromatin, hydrolysed with increasing concentrations of MNase, was analysed by gel electrophoresis and Southern blotting. The EcoRI hydrolysed genomic DNA (lanes 2 and 8), and the MNase treated chromatin was separated on a 1.3% agarose gel (lanes 3 to 7) and visualized with SybrSafe DNA stain. The gel was blotted and hybridized with the radioactively labelled, isolated tri-nucleosmal DNA (see Figure 1C) originating from the low-MNase fraction (3n lowMN probe; lanes 8 to 13). The sizes of the DNA marker (lane 1) are indicated. (**B**) A Southern blot as shown in (**A**), however, the blot was hybridized with the tri-nucleosomal DNA originating from the high-MNase fraction (3n highMN probe).

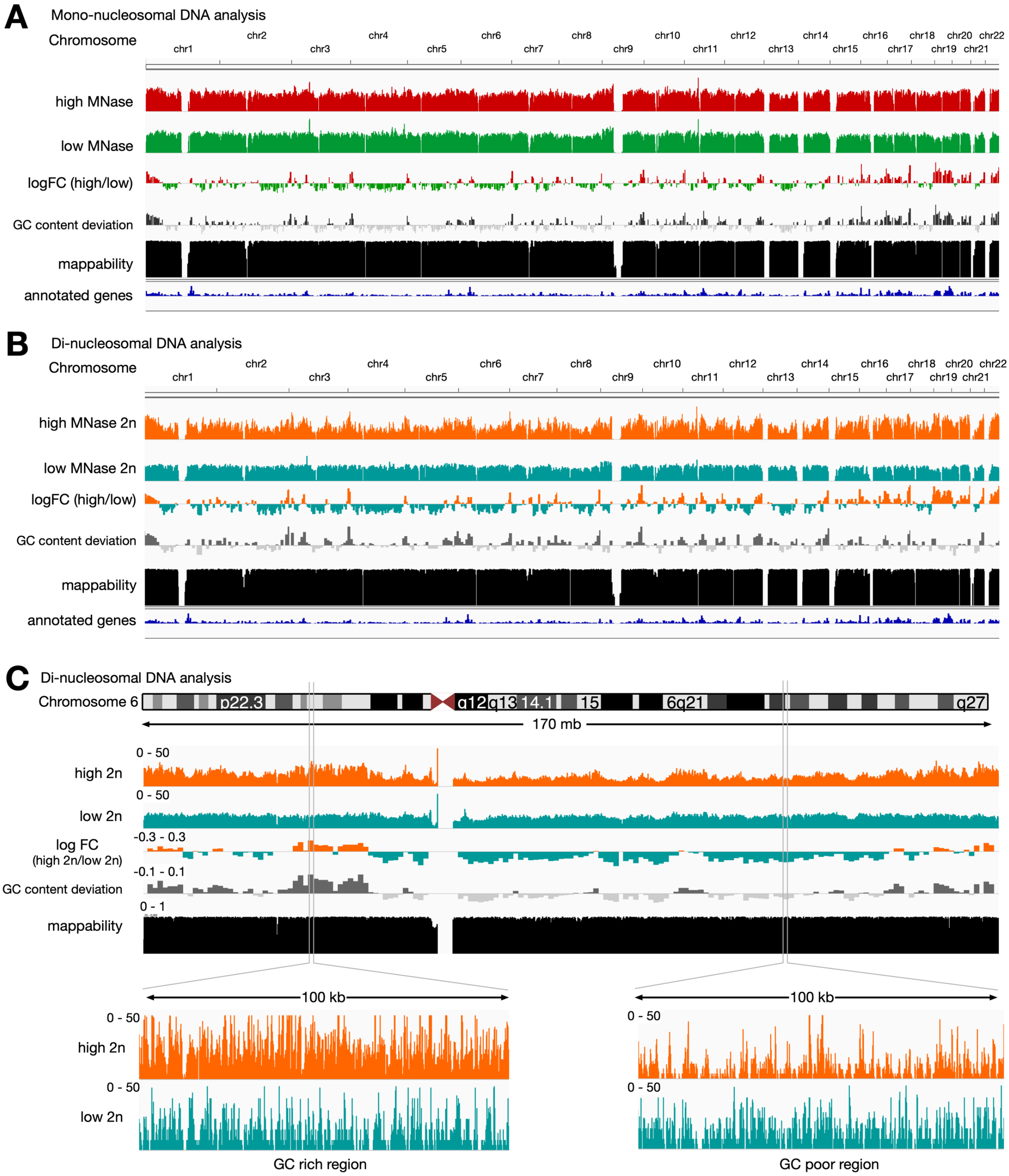

Supplementary Figure S4. Genome browser plot showing the nucleosome occupancy distribution of (**A**) mono-nucleosomal DNA and (**B**) di-nucleosomal DNA along the whole genome and (**C**) di-nucleosomal DNA along Chromosome 6. The GC content variation is displayed as the deviation from the genome wide average (40.5%).

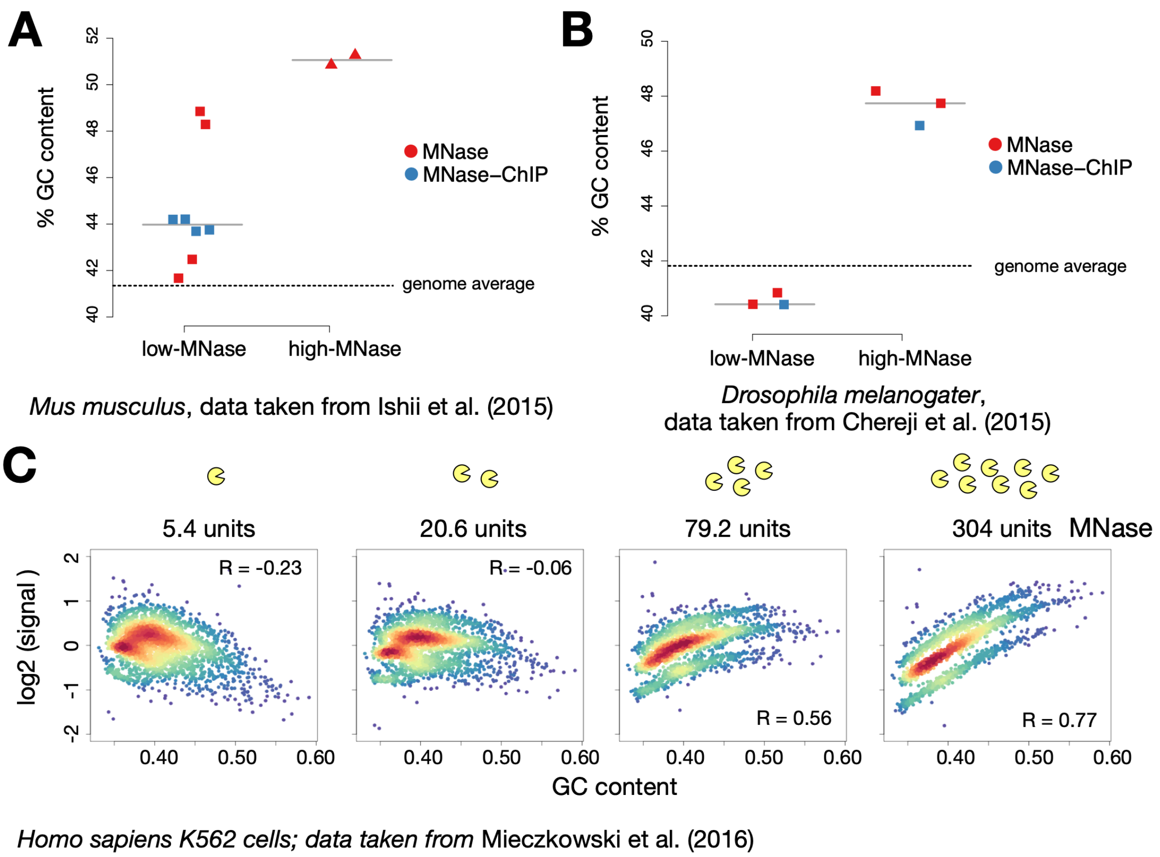

Supplementary Figure S5. Sequence preferences of MNase isolated nucleosomes from re-analysed published data sets, as indicated**.** (**A**) and (**B**) Average GC content of low- and high-MNase isolated fragments. Blue dots mark experiments that after MNase hydrolysis include an additional histone immunoprecipitation step (either H2B- or H3-ChIP). (**C**) Genome wide correlation of GC content and nucleosome occupancy in 1 Mb windows. Chromatin digestions using different MNase concentrations, as indicated, are shown.

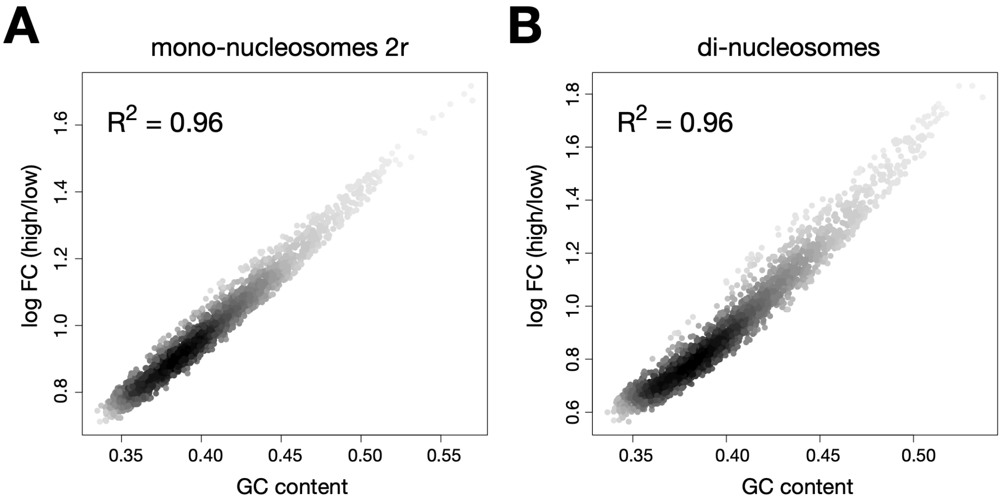

Supplementary Figure S6 Genome wide correlation of GC content and nucleosome occupancy profiles. Dots represent 1 Mb non-overlapping windows; The coefficient of determination (R^2) refers to the result of a simple linear regression between GC content and the log FC of high-MNase versus low-MNase of (**A**) the mono-nucleosomal DNA from independent biological replicates and (**B**) di-nucleosomal DNA.

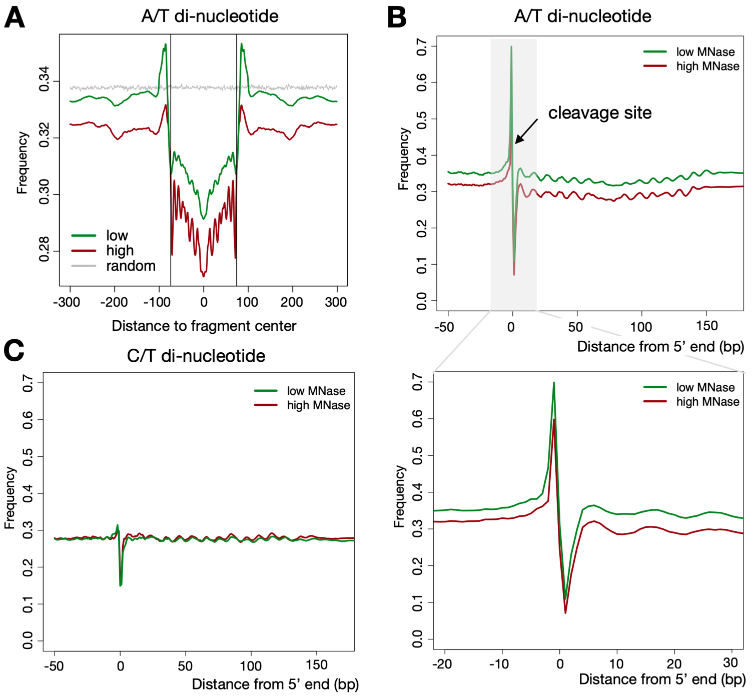

Supplementary Figure S7. Nucleotide frequencies relative to high- and low-MNase extracted mono-nucleosomal fragements. (**A**) Average A/T (AA, AT, TA, TT) – di-nucleotide frequency centred on mapped mono-nucleosomal fragment midpoints. A random distribution (grey line) was simulated on 1 million randomly generated fragments of 147 bp lengths. Vertical lines indicate nucleosome boundaries (± 73 bp). (**B**) A/T and (**C**) C/T di-nucleotide frequencies relative to MNase cleavage sites (5' end of read).

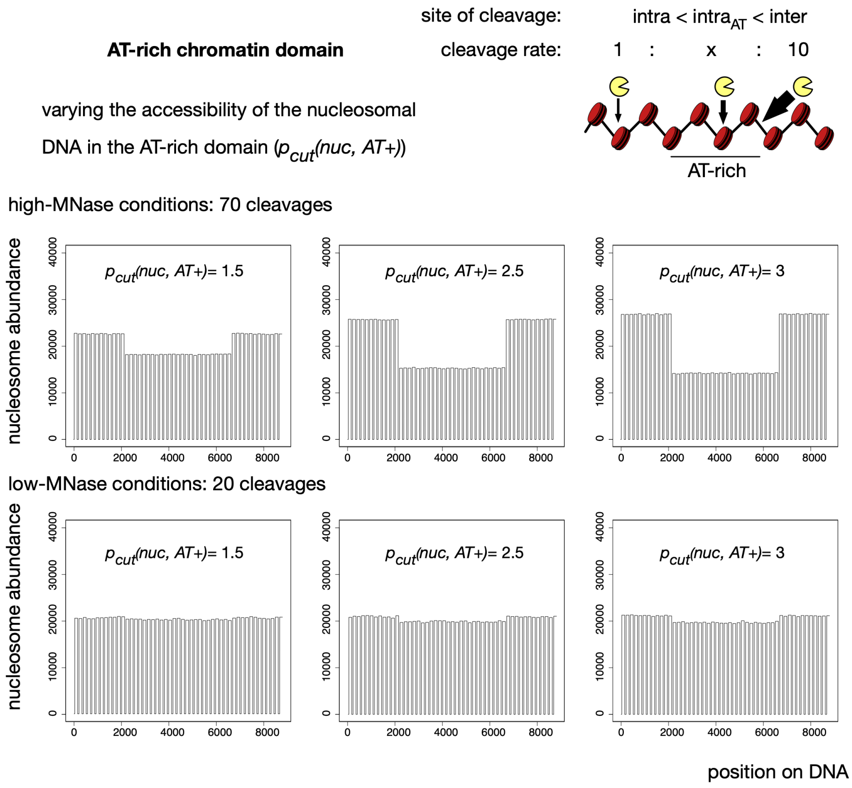

Supplementary Figure S8. Simulation of high- and low-MNase digestion with varying accessibility of the nucleosmal DNA in the AT-rich domain. Used cleavage probabilities: $p_{cut}(nuc, AT+) = x \cdot p_{cut}(nuc)$ and $p_{cut}(linker) = 10 \cdot p_{cut}(nuc)$.

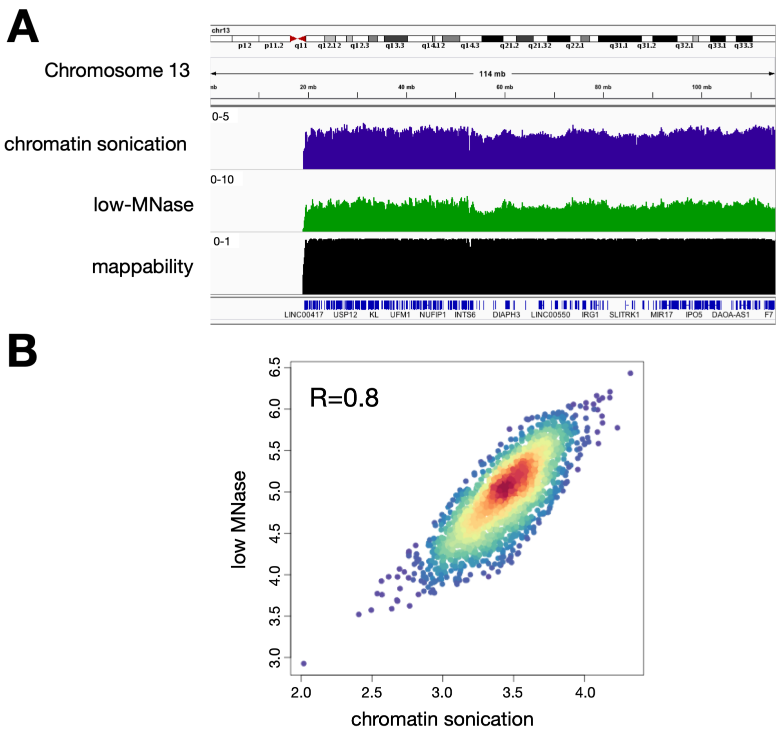

Supplementary Figure S9. Comparison of low-MNase digestion and sonication of chromatin. (**A**) Genome browser plot showing the average read count of sonicated chromatin (top track) and of low-MNase extracted mono-nucleosomal fragments (middle track) along Chromosome 13. (**B**) Genome wide correlation of low-MNase and chromatin sonication. Dots represent 1 Mb non-overlapping windows. Single-end reads obtained after sequencing of sonicated chromatin were extended to 150-bp to match the average mono-nucleosome sized fragments of low-MNase digestion.

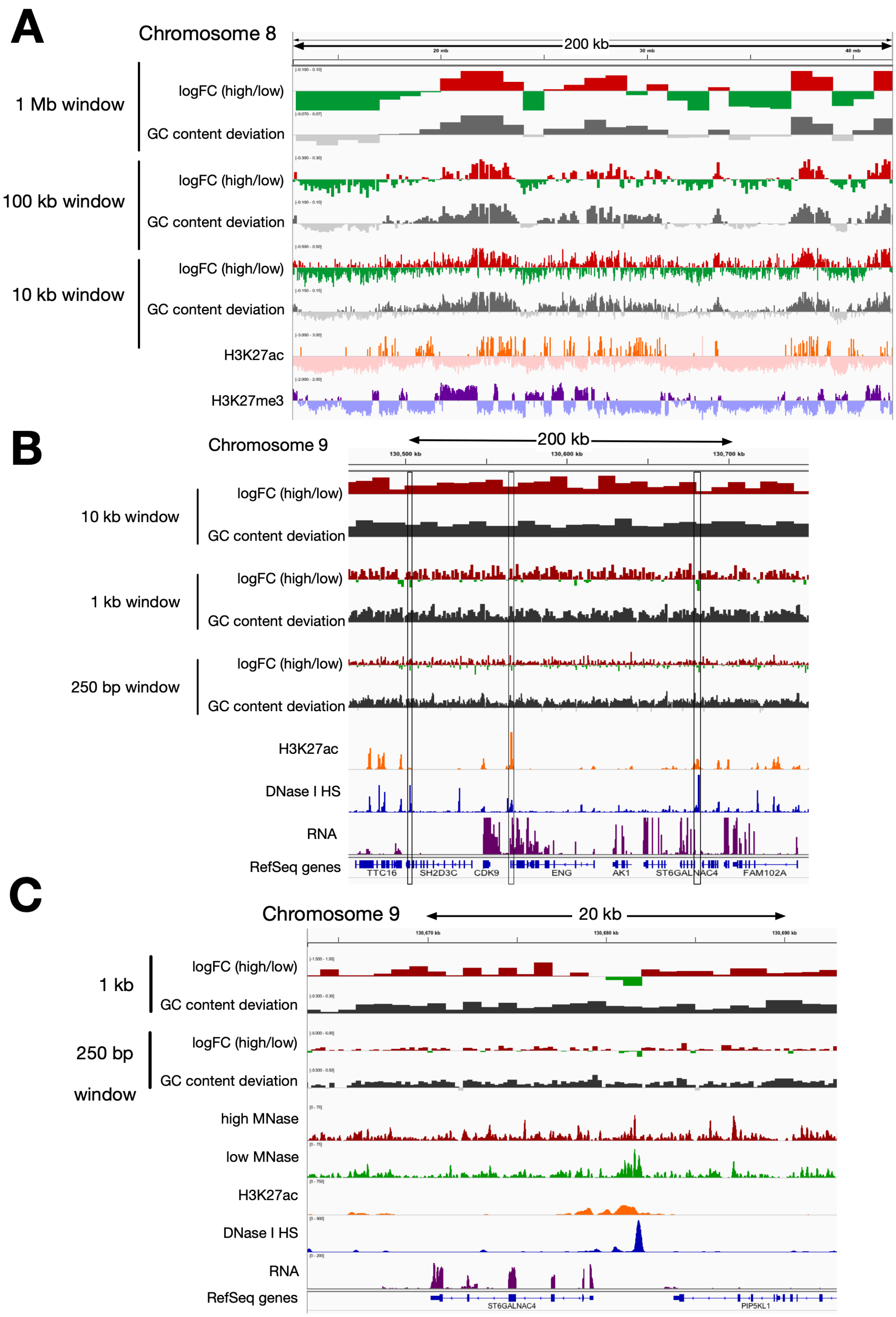

Supplementary Figure S10. Genome browser snapshots showing varying resolutions of differential MNase signal and GC content. Window sizes used are indicated on the left side. (**A**) H3K27me3 (ENCODE, ENCFF958BAN) and H3K27ac (ENCODE, ENCFF311EWS) are displayed as logFC over the input signal. (**B**) Rectangles highlight local accessible sites marked by DNase I HS (ENCFF567PRQ) and H3K27ac (ENCFF311EWS) enrichment. The RNA track (ENCSR000EYQ) was downloaded from ENCODE repository. (**C**) Detailed view of an intergenic hyperaccessible site marked with H3K27ac.

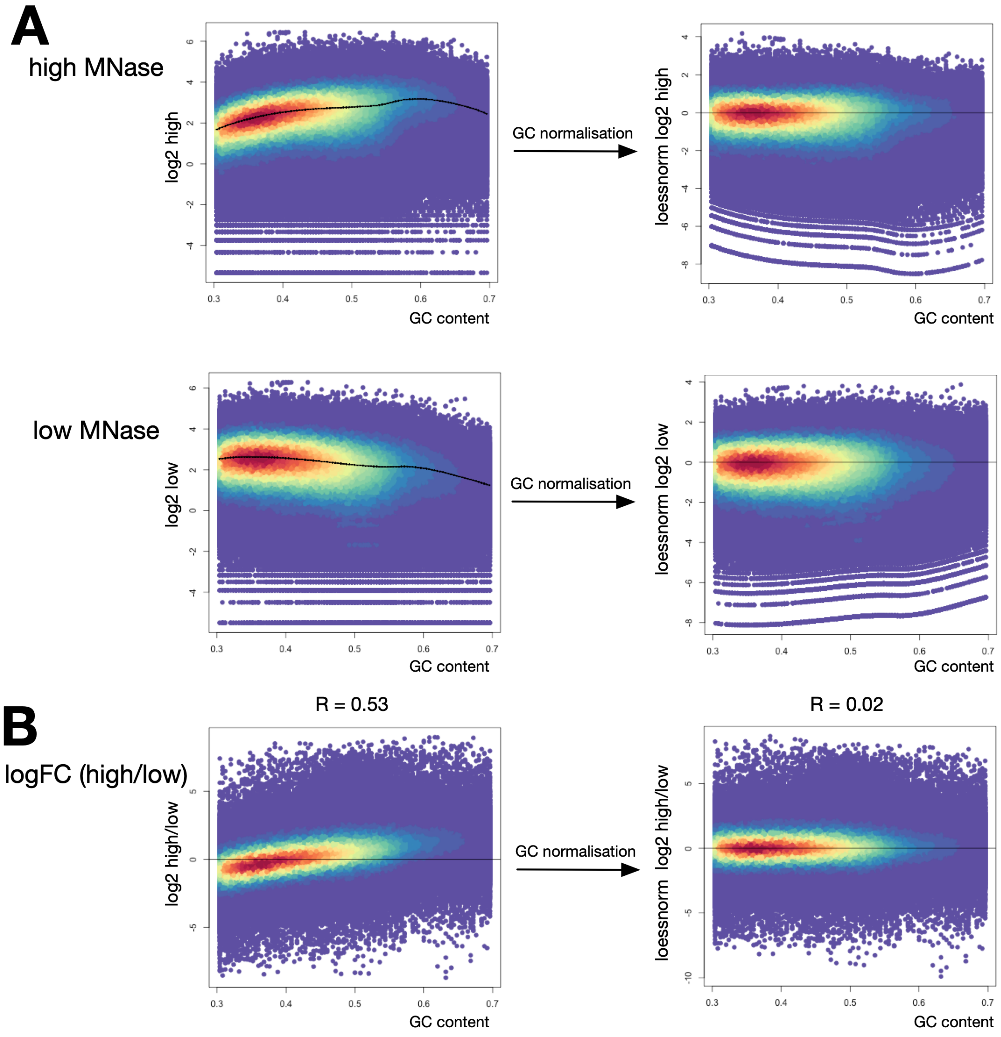

Supplementary Figure S11. LOESS GC normalisation of high- and low-MNase data. (**A**) The genome was partitioned into 250 bp bins, and the average nucleosome profile of low- / high-MNase and the GC content was calculated. Next, a LOESS curve was fitted to the data (left panel), which was then used to normalize for the GC bias (right panel). (**B**) The dependency of the MNase logFC to the GC content before and after individual normalization of low- / high-MNase profiles.

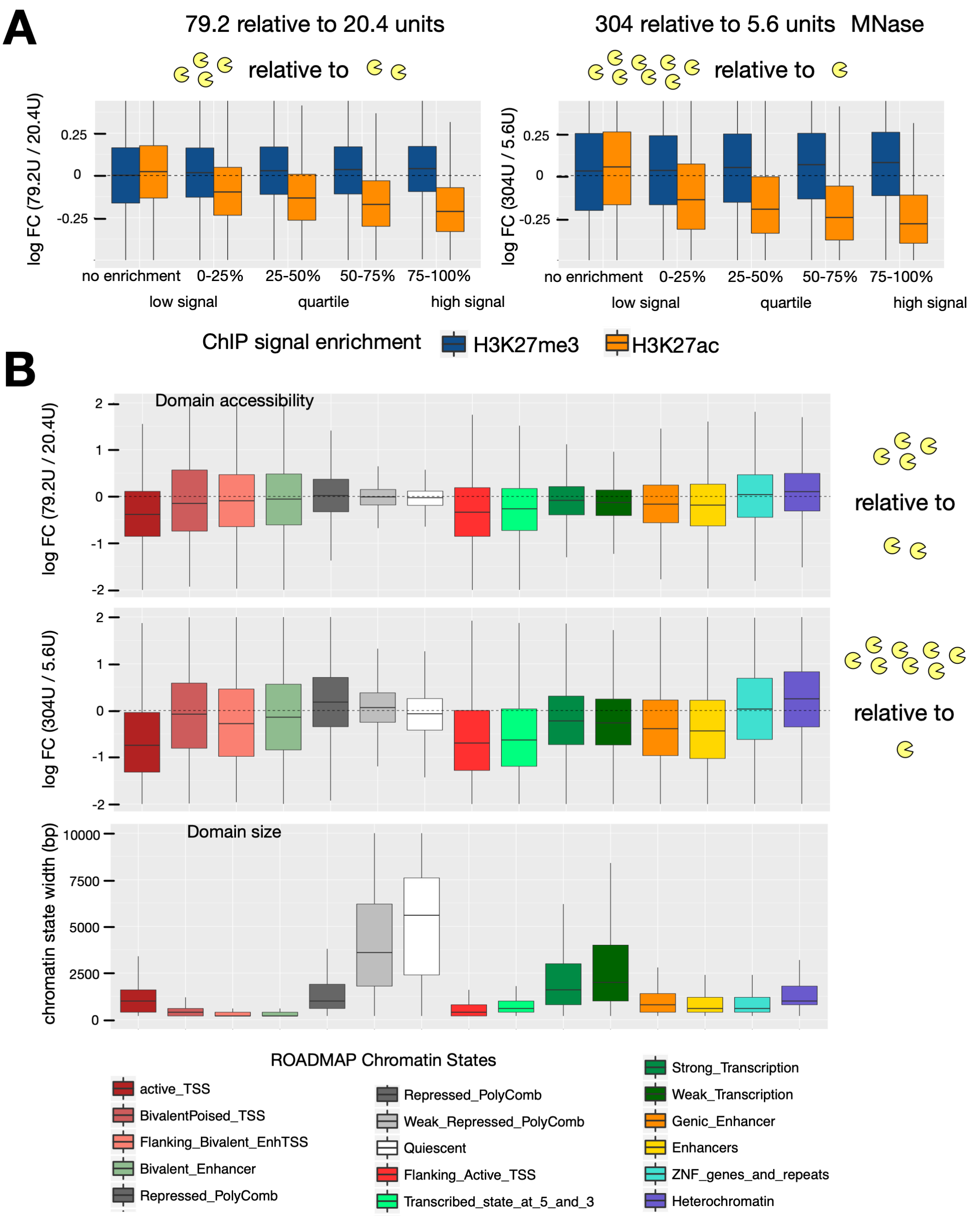

Supplementary Figure S12. Comparison of varying MNase concentrations to measure chromatin accessibility within annotated chromatin regions. Chromatin digestions in K562 cells using four distinct MNase concentrations (5.6U, 20.4U, 79.2U and 304U; Mieczkowski et al., 2016) were re-analysed. MNase sequence preferences were corrected by applying a LOESS normalization in 500 bp windows (as shown in Supplemental Figure S11). Differential nucleosome extraction was calculated as the log2 fold change in nucleosome occupancy comparing two distinct MNase conditions, as indicated. (**A**) Differential nucleosome enrichment at H3K27me3 (blue; accession ENCFF958BAN) / H3K27ac (orange; accession ENCFF311EWS) enriched regions. The average MNase signal was calculated in 10 kb non-overlapping windows spanning the whole genome. Windows were grouped on the basis of the ChIP enrichment signal into windows showing no ChIP enrichment (ChIP-signal < input signal) and windows enriched for the histone modification were further subdivided into quartiles of signal enrichment. (**B**) Differential MNase signal at annotated chromatin states (ROADMAP; accession E123).

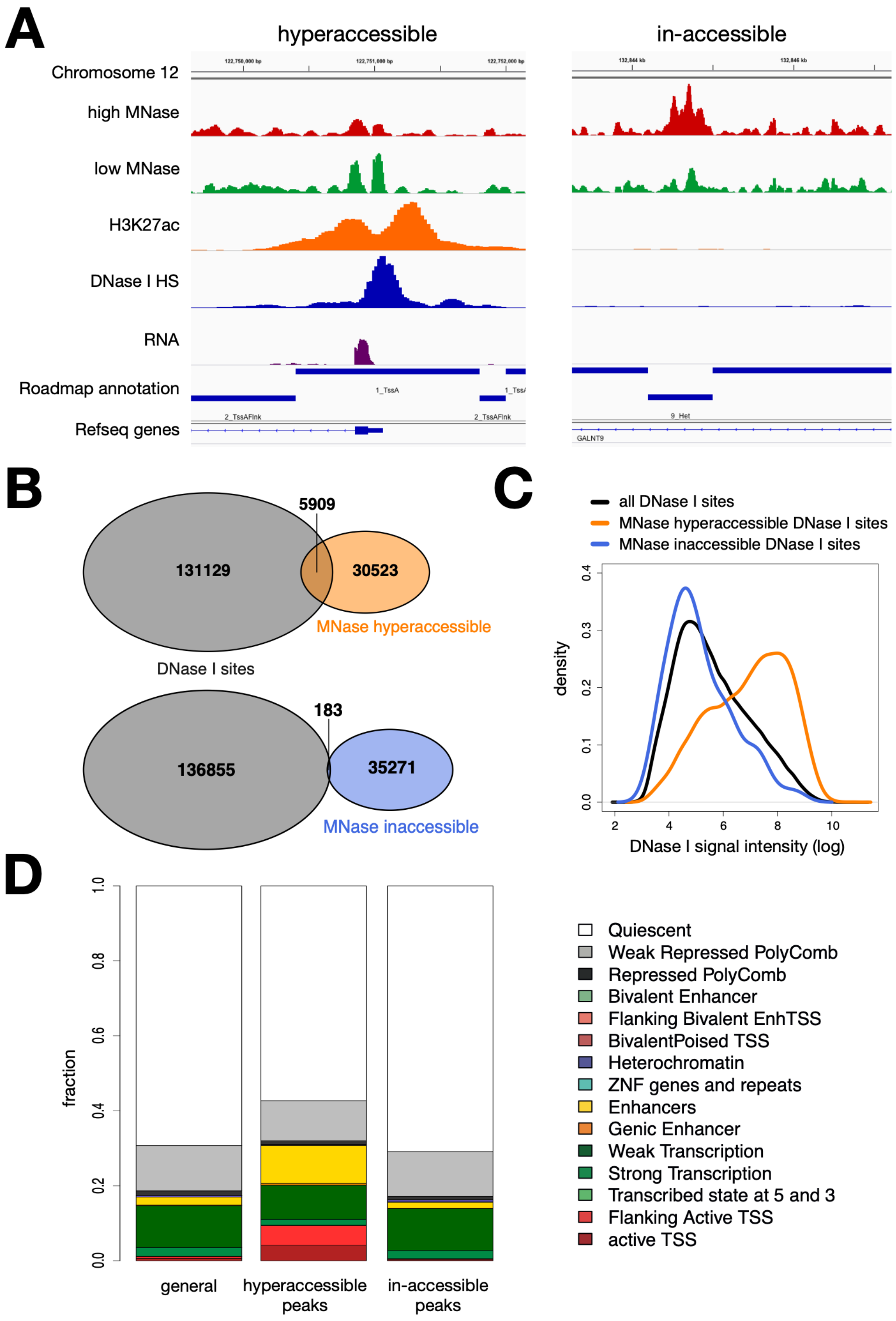

Supplementary Figure S13. Local sites of differential MNase enrichment. (**A**) Local sites of either low-MNase enrichment (left panel) or high-MNase enrichment (right panel). (**B**) Co-occurrence of annotated DNase I sites (ENCODE, ENCFF692NCU) with called hyperaccessible sites (low-MNase enrichment) and in-accessible sites (high-MNase enrichment). (**C**) Density of DNase I signal in all annotated peaks (black), peaks coinciding with in-accessible (blue) or hyperaccessible (orange) MNase sites. (**D**) Functional genome annotation of differential MNase sites. Barplots showing the fraction of chromatin states (ROADMAP, HeLa E115) assigned to hyperaccessible and inaccessible sites.
